# Supplementary material for: HIV-1 Uncoating and Nuclear Import Precede the Completion of Reverse Transcription in Cell Lines and in Primary Macrophages
Source: Viruses. 2020 Oct 30;12(11):1234. doi: 10.3390/v12111234 (PMC7693591; doi:10.3390/v12111234)
Supplement: Supplementary file 1 [file viruses-12-01234-s001.zip › Videos/Viruses MDM_Supplementary Figures-movies.pdf]

Supplementary Figure Legends

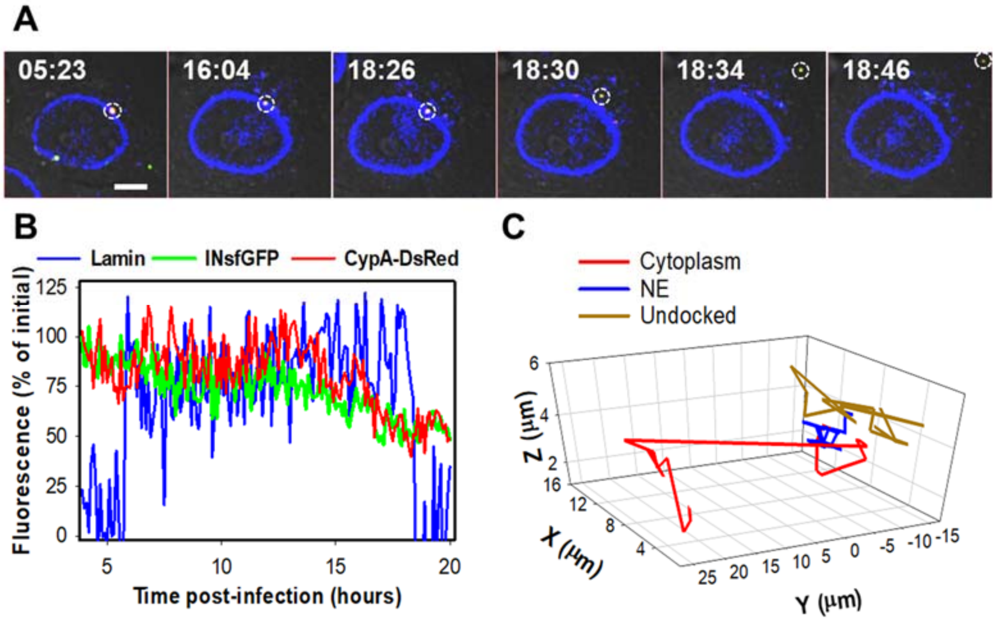

Suppl. Fig. S1. Failure to lose CDR at the nuclear envelope results in undocking of HIV-1 cores. Images (A), fluorescence intensity traces (B) and single particle trajectory (C) of the HIV-1 INsfGFP/CDR labeled core marked by dashed lines in (A) which was docked for more than 13 h at the nuclear envelope labeled with EBFP2-lamin (blue), but did not loose a large portion of its CDR signal (red). The core eventually moved back into the cytoplasm (undocking). A single particle trajectory shown in (C) is color-coded: cytoplasmic track before docking (red), docked (blue) and undocked (brown). Scale bar is 5 µm.

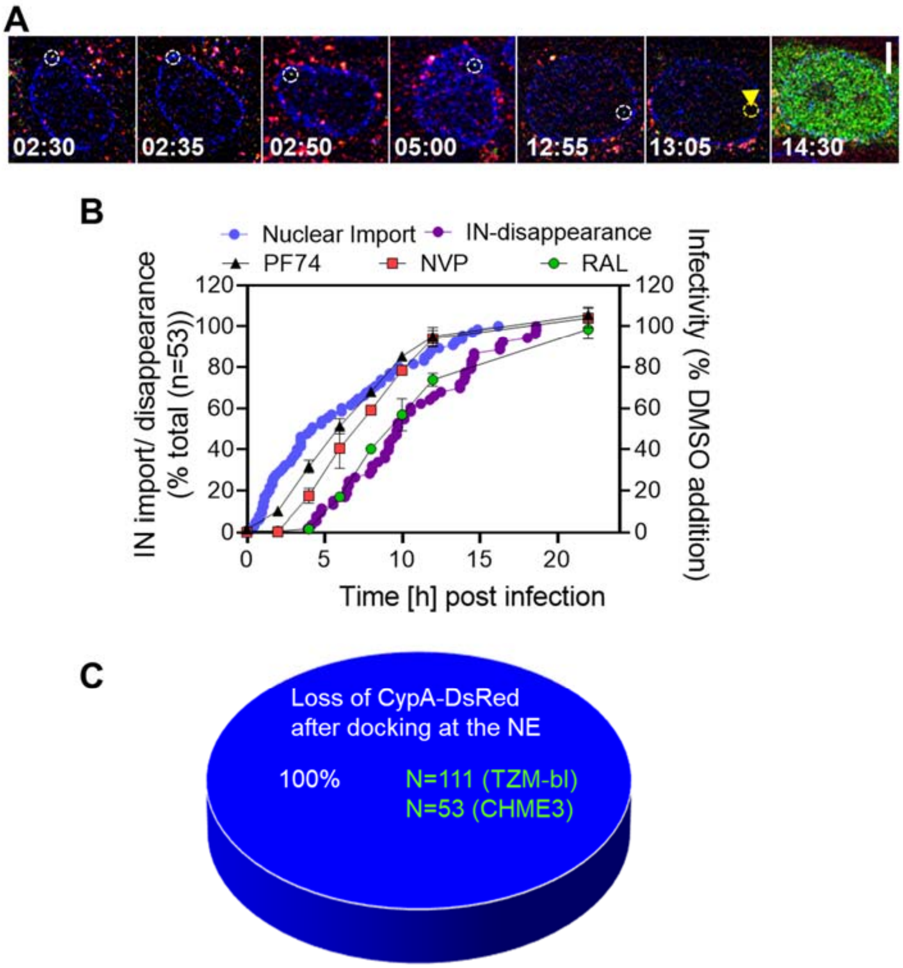

Suppl. Fig. S2. The time courses of HIV-1 nuclear import, completion of vDNA synthesis and integration in CHME3 cells. (A) Images of CDR containing HIV-1 core docked at the nuclear envelope labeled with EBFP2-Lamin (blue) of CHME3 cells at 2h 30m. Following a loss of CDR signal (2h 35m), INsfGFP (green) labeled VRCs enter the nucleus (2h 50m), traffic for ~10 hours, and disappear (13h 05m; marked by yellow dashed lines and arrowhead, respectively) prior to the expression of the eGFP reporter of infection (14h 30m). (B) The kinetics of nuclear import (blue circles) and INsfGFP disappearance (purple circles) in CHME3 cells obtained by live-cell imaging, as shown in (A). A total of 63 nuclear import and disappearance events are shown. The kinetics of completion of reverse transcription and integration were determined in parallel experiments by adding NVP (10 μM) or RAL (10 μM) at indicated time points after infection. The time-course of HIV-1 escape from PF74 (2 μM) is also plotted. The resulting infection measured by luciferase expression (normalized to DMSO) is plotted on the right axis. Error bars are means and SEM from 3 independent time-of-drug addition experiments. The nuclear import and INsfGFP disappearance kinetics are plotted as cumulative data from 3 independent experiment. (C) A summary of uncoating events (loss of CDR) at the nuclear membrane followed by nuclear import of INsfGFP complexes leading to in TZM-bl (n=111) and CHME3 cells (n=53) from 3 independent experiments performed at low MOI of 0.2.

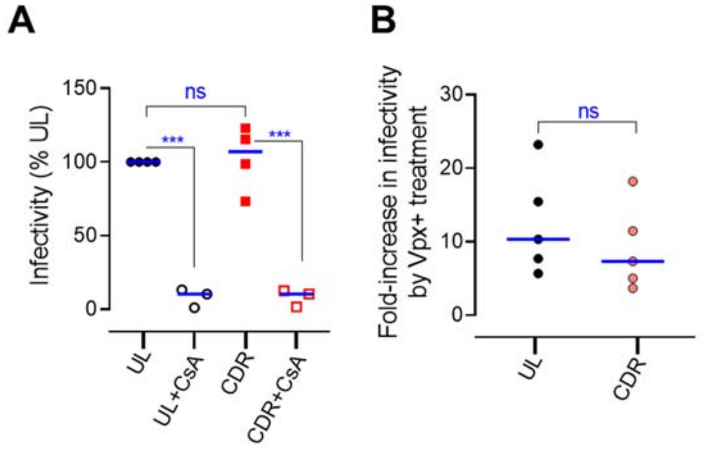

Suppl. Fig. S3. CDR-labeling does not affect HIV-1 infectivity in MDMs. (A, B) Single-round infectivity of VSV-G pseudotyped NL4.3 R-E- Luc HIV-1 unlabeled (UL) or labeled with CypA-DsRed (CDR) was determined in untreated (A) or untreated and Vpx(+) treated (B) MDMs at 72 hpi (MOI of 1). CsA (2.5 µM) was added at the time of synchronized infection in (A) to test the effect of CypA displacement from the virus core on infectivity. Infectivity was normalized to UL control in (A). The fold-increase in infectivity in Vpx(+) treated over untreated cells is plotted in (B). The mean values (blue lines) are from triplicate experiments from 4 donor in (A) and 5 donors in (B)).

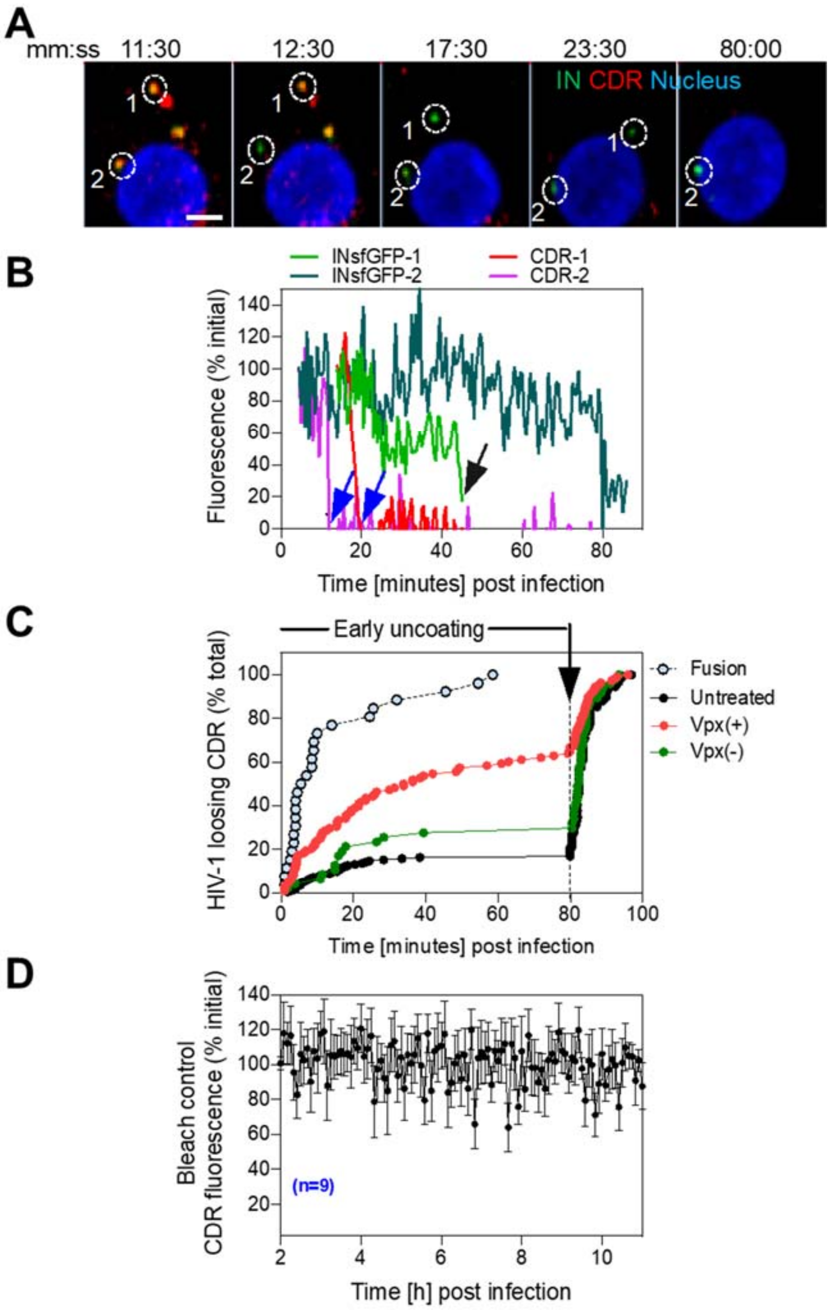

Suppl. Fig. S4. SAMHD1 depletion in MDMs accelerates early cytoplasmic HIV-1 uncoating. Untreated or SIV-Vpx(+)-VLP treated MDMs were infected at MOI 0.008 (measured in TZM-bl) with VSV-G pseudotyped HIV-1 colabeled with INsfGFP and CDR. (A) Images and (B) fluorescence intensity traces of single particles showing an abrupt loss of CDR from INsfGFP labeled complexes within 80 min post-infection (termed early uncoating). Fluorescence intensity traces for two uncoating cores marked 1 and 2 in images (A) are shown in (B). Blue and black arrows in (B) point to the time of uncoating of the two cores and cytoplasmic disappearance of VRC-1, respectively. Scale bar in (A) is 5 μm. (C) The kinetics of early uncoating was visualized up to 2 hrs post-infection. CsA (10 μM) was added to cells at the time of imaging to visualize virus fusion or at 80 min post-infection (arrow) to displace CDR from viral cores and enable quantification of post-fusion INsfGFP-labeled cores that contained robust p24/CDR signal. (D)

Fluorescence intensity of cores bound on cover glass and imaged using the exact same conditions as live cell imaging (photobleaching control). Error bars in (D) represent STD from n=9 cores. Note that a subset of cores tracked in the related Figure 3D shows only borderline decrease (<20%) in CDR fluorescence, similar to photobleaching control shown here.

Supplementary Movie legends

Suppl. Movie S1. Live-cell imaging of productive HIV-1 nuclear import that results in integration and eGFP reporter expression in TZM-bl cells. Cells were infected at low MOI 0.2 with VSV-G pseudotyped HIVeGFP virus co-labeled with INsfGFP and CDR, the markers of VRCs and CA, respectively, and imaged for 22 h. Movie shows a single INsfGFP (green) and CDR (red) labeled virus arriving at the nuclear envelope (EBFP2-Lamin, blue), uncoating (loss of CDR) and entering the nucleus at 4.5 h. Following nuclear import, the productive IN-labeled VRC (marked by arrow) traffics for several hours prior to its disappearance at 10 hpi, which correlates with integration and subsequent eGFP reporter expression at 14 hpi. Note that the non-integrating nuclear VRCs do not disappear by the time of eGFP expression (marked by arrows at 10 hpi). Scale bar is 5 μm. (see also related panels in Fig. 1A)

Suppl. Movie S2. Lack of uncoating at the NE results in undocking of viral cores. TZM-bl cells were infected with VSV-G pseudotyped HIVeGFP virus co-labeled with INsfGFP and CDR at low MOI 0.2 and imaged for 22 h. Movie starts at 4h and shows an example of a single INsfGFP (green) and CDR (red) labeled virus docked at the nuclear envelope (EBFP2-Lamin, blue) for over 11 hours without losing CDR. Lack of uncoating of this core results in undocking (at 13 h) and anterograde trafficking to the cellular periphery. Scale bar is 5 μm. (see also related panels in Suppl. Fig. 1).

Suppl. Movie S3. Live-cell imaging of the productive HIV-1 nuclear import that results in integration and eGFP reporter expression in CHME3 cells. Cells were infected with VSV-G pseudotyped HIVeGFP virus co-labeled with INsfGFP and CDR at low MOI 0.2 and imaged for 22 h. Movie starts at 2h 30m and shows a single INsfGFP (green) and CDR (red) labeled virus already docked at the nuclear envelope (EBFP2-Lamin, blue) undergoing uncoating (loss of CDR) and nuclear import (at 1.5 h) followed by INsfGFP signal disappearance in the nucleus after several hours and eGFP reporter expression (at 12 h). Scale bar is 10 μm. (see also related panels in Suppl. Fig. 2A).

Suppl. Movie S4. A large fraction of post-fusion cores remains stable in the cytoplasm of untreated MDMs. MDMs were infected with VSV-G pseudotyped HIVeGFP virus co-labeled with INsfGFP and CDR at low MOI 0.5 and imaged between 0 and 2 hpi. The movie shows several INsfGFP (green) and CDR (red) co-labeled cores trafficking in the MDM cytoplasm without losing CDR signal up to 1h 20m. Addition of CsA at this time point reveals post-fusion cores based on the loss of CDR signal without changing the INsfGFP signal. Scale bar is 10 μm. (see also related panels in Fig. 3A).

Suppl. Movie S5. Treatment of MDMs with Vpx(+) containing SIV-VLPs promotes early cytoplasmic uncoating and reduces the fraction of stable post-fusion cores. MDMs treated with Vpx(+) were infected with VSV-G pseudotyped HIVeGFP virus co-labeled with INsfGFP and CDR at low MOI 0.5 and imaged

between 0 and 2 hpi. The movie shows several INsfGFP (green) and CDR (red) co-labeled cores trafficking in MDM cytoplasm and uncoating prior to CsA addition at 80 min. The movie pauses at 1h 20m to show the number of INsfGFP-positive/CDR-positive cores prior to CsA addition. Only a few post-fusion cores are detected to loose CDR after CsA addition. Scale bar is 10 µm. (see also related panels in Fig. 3A).

Suppl. Movie S6. HIV-1 cores retain CDR-CA marker for several hours in Vpx(+) treated MDMs. MDMs treated with Vpx(+) were infected with VSV-G pseudotyped HIVeGFP virus co-labeled with INsfGFP and CDR at low MOI 0.5 and imaged between 2 and 18 hpi. The movie shows high-mobility of MDMs across the field of view. Also shown, is the trafficking of long-lived INsfGFP (green) and CDR (red) labeled HIV-1 cores for several hours. Note that only co-labeled cores appear to survive trafficking in MDM cytoplasm by the end of movie. The movie is a stitched view of 4 neighboring fields of view. Scale bar is 10 µm. (see also related panels in Fig. 3C).

Suppl. Movie S7. Live-cell imaging of the HIV-1 nuclear import in Vpx(+) MDMs. MDMs were infected with VSV-G pseudotyped HIVeGFP virus co-labeled with INsfGFP and CDR at low MOI 0.5 and imaged for 22 h. The movie shows single INsfGFP (green) and CDR (red) labeled virus docking at the nuclear envelope at 1.2 h (Hoechst, blue), uncoating (loss of CDR) at 1.4 h, nuclear import at 1.6 h, and intra-nuclear trafficking. Scale bar is 10 µm. (see also related panels in Fig. 4A).
